# Health-related quality of life, anxiety and depression in young adults with disability benefits due to childhood-onset somatic conditions

**DOI:** 10.1186/1753-2000-7-12

**Published:** 2013-04-15

**Authors:** Eefje Verhoof, Heleen Maurice-Stam, Hugo Heymans, Martha Grootenhuis

**Affiliations:** 1 Psychosocial Department, Emma Children’s Hospital, Academic Medical Center, University of Amsterdam, Amsterdam, the Netherlands; 2 Department of Pediatrics, Emma Children’s Hospital, Academic Medical Center, University of Amsterdam, Amsterdam, the Netherlands

**Keywords:** Young adults, Chronic disease, Disability benefit, Health-related quality of life, Anxiety and depression, Work force participation

## Abstract

**Background:**

As the treatment of chronic or life-threatening diseased children has dramatically over recent decades, more and more paediatric patients reach adulthood. Some of these patients are successfully integrating into adult life; leaving home, developing psychosocially, and defining a role for themselves in the community through employment. However, despite careful guidance and support, many others do not succeed. A growing number of adolescents and young adults who have had a somatic disease or disability since childhood apply for disability benefits. The purpose of this study was to assess the health-related quality of life (HRQoL), anxiety and depression of young adults receiving disability benefits because of somatic conditions compared to reference groups from the general Dutch population and to explore factors related to their HRQoL, anxiety and depression.

**Methods:**

Young adults (N = 377, 22–31 yrs, 64.3% female) claiming disability benefits completed the RAND-36 and an online version of the HADS. Differences between respondents and both reference groups were tested using analysis of variance and logistic regression analysis by group and age (and gender). Regression analyses were conducted to predict HRQoL (Mental and Physical Component Scale; RAND-36) and Anxiety and Depression (HADS) by demographic and disease-related variables.

**Results:**

The respondents reported worse HRQoL than the reference group (−1.76 Physical Component Scale; -0.48 Mental Component Scale), and a higher percentage were at risk for an anxiety (29.7%) and depressive (17.0%) disorder. Better HRQoL and lower levels of anxiety and depression were associated with a positive course of the illness and the use of medical devices.

**Conclusions:**

This study has found worse HRQoL and feelings of anxiety and depression experienced by young adults claiming disability benefits. Healthcare providers, including paediatric healthcare providers, should pay systematic attention to the emotional functioning of patients growing up with a somatic condition in order to optimise their emotional well-being and adaptation to society during their transition to adulthood. Future research should focus on emotional functioning in more detail in order to identify those patients that are most likely to develop difficulties in emotional functioning and who would benefit from specific psychosocial support aimed at workforce participation.

## Background

Due to improved treatment possibilities and the positive consequences for life expectancy, the number of chronically ill children who live for longer is increasing, and more paediatric patients with somatic conditions are living into adulthood [1]. For these children, transition into adulthood is a critical phase. Children and adolescents with chronic illnesses are expected to go through similar developmental stages as their healthy peers; they will leave home, develop psychosocially, and define their role in the community through employment or other activities [2]. For patients with impairments, reaching these developmental stages can be challenging. Research findings indicate that school-aged children with chronic conditions, regardless of their diagnosis, are more limited in their participation in everyday life than their peers [3,4]. Also, research has showed that adolescents and young adults with disabilities often follow atypical developmental patterns when compared to their peers without a disability [5-7] and that they are at risk of poor educational, vocational and social outcomes in adulthood [3,8-10].

In the Netherlands, some 500,000 children (14%) are growing up with a chronic condition; 90% of them will reach adulthood [1]. As a result, many patients with a childhood-onset chronic condition will reach the age at which they enter the labour market. In the Netherlands, young people who are partially or fully incapable of working, due to a childhood-onset chronic condition, may be eligible for a benefit under the scheme for young disabled persons: Wajong (the Invalidity Insurance Act for Young Disabled Persons). The fact that young adults with Wajong benefits due to chronic conditions lag behind their peers in work experience is undesirable since employment is an important way to participate in social life. Besides money, employment offers many other additional immaterial advantages such as the possibility for self-development, social relationships, development of skills, daily routines, and, in many cases, meaning in life [11]. Consequently, employment has implications for the patients’ economic and social well-being in adulthood [12]. Furthermore, evidence shows that employment is often linked with higher levels of mental well-being in the general population [13].

However, few studies have focused on the emotional well-being of young adults with childhood onset chronic conditions who encounter barriers when pursuing employment, as compared to young adults without chronic conditions. Also, the HRQoL and emotional functioning of young adult beneficiaries with a childhood-onset somatic condition as a group has never been studied. Since they can be considered as the most vulnerable young adults with chronic conditions - those who have to apply for disability benefits as a result of their conditions - it is important to know to what extent the chronic conditions are considered a problem in daily life and affect their emotional well-being. Awareness for these problems is of utmost importance. Given the increase in the number of children and adolescents with a childhood-onset chronic condition and the growing number of them applying for disability benefits, it is essential to gain insight into their HRQoL and emotional functioning in order to be able to develop strategies to support this vulnerable population towards adulthood independence. Therefore, the purpose of this study was to assess the health-related quality of life (HRQoL), anxiety and depression of young adults claiming disability benefits because of somatic conditions compared to reference groups from the general Dutch population and to explore the relation of demographic and disease-related factors with their HRQoL, anxiety and depression. We hypothesized that young adults claiming disability benefits experience worse HRQoL and more anxiety and depression symptoms than reference groups from the general Dutch population.

## Methods

### Procedures

This study was conducted within the framework of a large cross-sectional study (EMWAjong), a study directed at investigating psychosocial functioning in young adults with a Wajong benefit for a childhood-onset chronic somatic condition and the factors affecting their vocational success. In this article we will refer to this group as ‘young adults claiming disability benefits’. All young adults between 22 and 31 years of age who claimed a Wajong benefit in the year 2003 or 2004 for a chronic somatic condition were invited to participate in EMWAjong via a letter. Participation meant completing an online questionnaire. Those with no sustainable work opportunities (classified as fully incapable for work) were excluded because the EMWAjong study aimed to identify factors that could help to improve vocational success. Those with serious cognitive impairment or psychiatric conditions were also excluded because the EMWAjong study was directed at young adults with childhood-onset somatic conditions.

In total, 2,046 persons were invited to take part in the study. To maintain the privacy of the beneficiaries, the invitation letter was sent by UWV, the Dutch benefits agency. The letter contained a personal log in code, a password and a link to the online questionnaire. After two weeks, participants received a reminder letter. Participants who completed the entire questionnaire received a gift voucher. The study was performed according to the regulations of the medical ethical committee; due to the once-only internet-based nature of the survey, no formal approval by the medical ethics committee was required.

### Measures

HRQoL was assessed using the RAND-36. The RAND-36 is a Dutch version of the MOS-SF-36 Health Survey and is almost identical to the Dutch SF-36 [14]. The RAND-36 is a multidimensional questionnaire consisting of 36 items with standardized response choices, clustered in 8 multi-item scales: Physical Functioning (PF), Social Functioning (SF), Role limitations owing to Physical health problems (RP), Role limitations owing to Emotional problems (RE), general Mental Health (MH), Vitality (VT), Bodily Pain (BP), and General Health perceptions (GH). All raw scale scores were converted to a 0–100 scale, with higher scores indicating higher levels of functioning or well-being. The validity and reliability of the RAND scales were satisfactory [15]. Among the EMWAjong group we found Cronbach’s alphas of 0.75 to 0.95. Overall physical and mental health was assessed by aggregating all scale scores according to the algorithm described by Ware and Kosinski [16], yielding the so-called Physical Component Scale (PCS) and to the Mental Component Scale (MCS). The weights of the scales were derived from a Principal Components Analysis with the RAND-36 data of a Dutch reference group [17], using a non-orthogonal rotation (Oblimin), based on the assumption that physical health and mental health are interdependent. A Dutch reference group was used comprising peers from the general population. This reference group was recruited through general practitioners for a previous study on late psychosocial consequences of cancer in childhood (see Stam et al. 2005 for details [7]). The reference sample consisted of 508 respondents, 239 men (47.0%) and 269 women (53.0%). Mean age was 24.2 years (SD 3.8, range 18.0–30.9).

Anxiety and depression were measured using the Hospital Anxiety and Depression Scale (HADS). This 14-item scale describes a 7-item depression scale, a 7-item anxiety scale and a total scale. The 14 items are scored on a four-point scale (0–3), producing a total score ranging from 0 to 21. Higher scores indicate more anxiety or depression symptoms in the past week. A score of 8 or above is generally used as a cut-off score and is considered indicative of a possible presence of a depression or anxiety disorder; a score of 8 or above is called *at risk*[18]. The Dutch version of the HADS showed satisfactory validity and reliability [19]. In this study, the internal consistency (Cronbach’s alpha) of the anxiety scale was 0.83 and of the depression scale 0.75. The data of the Dutch HADS reference group are available, collected by a research institute that is specialized in online survey research [20]. The HADS reference group consisted of 182 respondents from the general Dutch population, 69 men (37.9%) and 113 women (62.1%). Mean age was 27.1 years (SD 2.5, range 22.0–30.0).

Due to privacy reasons, no information about the chronic conditions of the participants was provided by the benefits agency. This information was therefore derived through beneficiaries’ self reports. The questions concerning the disease characteristics were chosen based on existing questionnaires [21] and recommendations from experts in the field. The following dichotomous disease-related variables were used in the present study: congenital disorder (yes/no), visible disease/disability (yes/no), the nature of the disease process over time (“course of disease”: stable or positive vs negative or variable), daily use of medication (yes/no), need for medical devices in daily life, e.g. hearing aid and wheelchair (yes/no), limitations in use of fingers/hands, sight, hearing, and not being able to sit/stand for half an hour (yes/no).

### Statistical analysis

The Statistical Package for Social Sciences (SPSS) Windows version 16.0 was used for all the analyses. Gender and age differences between EMWAjong and both reference groups were tested with Chi2-tests and *t-*tests respectively. Age and gender distribution in the EMWAjong group differed significantly from the RAND-36 reference group; further analyses concerning HRQoL were therefore corrected for age and gender. In the case of the HADS analyses, correction for age was required, but not for gender.

Univariate analysis of variance (ANOVA) by group, age and gender was performed to test differences in HRQoL (mean scale scores) between EMWAjong and the RAND-36 reference group. ANOVA by group and age was performed to test differences on Anxiety and Depression (mean scale scores) between EMWAjong and the HADS reference group. Effect sizes (d) were calculated by dividing the difference in mean scale scores of the EMWAjong group and the reference group by the standard deviation of the scores in the reference group. We considered effect sizes up to 0.2 to be small, effect sizes up to 0.5 to be moderate and effect sizes up to 0.8 to be large [22].

In addition, logistic regression analyses by group and age were conducted in order to test whether the proportion of young adults that were *at risk* of an anxiety or depression disorder in the EMWAjong group differed from the proportion in the HADS reference group, using the odds ratios (OR) for group.

Finally, regression analyses were performed to predict HRQoL, as expressed by the Mental and Physical Component Scale of the RAND-36 (MCS, PCS), and Anxiety and Depression of the HADS, by demographic (age and gender) and disease-related variables (congenital disorder, visible disease/disability, course of the disease and medical devices). In line with Cohen [22], binary-coded variables of 0.3 were considered small, 0.5 medium and 0.8 large. For continuous variables, regression coefficients of 0.1 were considered small, 0.3 medium and 0.5 large.

A significance level of 0.05 was used for all analyses.

## Results

### EMWAjong group

A total of 415 young adults with a chronic somatic condition participated in the study (response rate 20.1%). Non-responders differed from responders with respect to gender; 51.4% vs. 64.3 % women (p < 0.05).

Thirty-nine respondents were removed from the analyses because of missing data on the RAND-36 questionnaire. In the case of the HADS, 38 respondents were removed. Consequently, the data of 376 and 377 participants respectively were used for the analyses of HRQoL and anxiety and depression: the group comprised 242 women (64.4 %) and 134 men (35.6%). The characteristics of the EMWAjong group are listed in Table 1.

**Table 1:** Demographic and medical characteristics of the EMWAjong group

**EMWAjong group (N = 376)**^**1**^			
	**M**	**SD**	**Range**
*Age at study (years)*	25.0	2.1	22.5 - 30.9
	**N**	**%**	
Gender			
*Female*	242	64.4	
*Male*	134	35.6	
**Chronic conditions**		**N**	**%**
Visually impaired/blind		58	14.3
Spasm		49	12.0
Rheumatoid arthritis		46	11.3
CFS/migraine		44	10.8
Hearing impaired/deaf		34	8.4
Epilepsy		34	8.4
Back complaints		31	7.6
Intestinal complaints		24	5.9
Lung complaints		21	5.2
Accident damage		21	5.2
Cancer		20	4.9
Paralysis		19	4.7
Muscular dystrophy		17	4.2
Arthritis		17	4.2
Kidney diseases		15	3.7
Skin disease		9	2.2
Heart disease		7	1.7
Liver disease		6	1.5
Other		127	31.0
**Disease characteristics**		**N**	**%**
Congenital disorder		211	50.8
*Visible disability*		171	42.0
Course of the disease			
- *Better*		71	17.4
*- Worse*		73	17.9
*- Variable*		93	22.9
- Constant		170	41.8
Daily medicine use		209	51.4
Medical devices		195	47.9
*Limitations in fingers/hand*		164	40.3
*Limitation of sight*		96	23.6
*Limitations of hearing*		35	8.6
*Able to sit half an hour*		377	92.6
*Able to stand half an hour*		241	59.2

^1^ Based on the number of respondents who completed both the RAND-36 and the HADS.

There were significant differences with respect to age and gender between the EMWAjong group and the RAND-36 reference group (p < 0.001). The EMWAjong group and the HADS reference group were significantly different with respect to age (p < 0.001).

### Health-related quality of life

The results of the ANOVA showed lower HRQoL for the EMWAjong group than the reference group on all domains (p < 0.001), except for General Mental Health (Table 2). Effect sizes ranged from −0.32 for Role limitations due to Emotional problems to −2.14 for Physical Functioning. The ANOVA for the Physical and Mental Component Scale confirmed these findings: the EMWAjong group scored significantly lower than the reference group, with effect sizes of −1.76 and −0.48 respectively.

**Table 2:** HRQoL (RAND-36) of the EMWAjong group versus the RAND-36 reference group; Mean scores, SD and effect sizes

	**EMWAjong group N = 376**	**RAND-36 reference group N = 508**		
			**F**	**Effectsize**
*Physical Functioning*			372.63*	−2.14
*Mean*	62.6	93.0		
*SD*	30.7	14.2		
*Social Functioning*			115.48*	−0.86
*Mean*	71.1	87.2		
*SD*	23.8	18.7		
*Role limitations Physical*			160.69*	−1.12
*Mean*	55.8	86.6		
*SD*	41.4	27.5		
*Role limitations Emotional*			16.69*	−0.32
*Mean*	77.8	87.2		
*SD*	36.1	29.0		
*General Mental Health*			3.88	−0.15
*Mean*	73.5	75.8		
*SD*	19.4	15.4		
*Vitality*			35.85*	−0.51
*Mean*	56.2	64.9		
*SD*	22.5	17.0		
*Bodily Pain*			75.57*	−0.75
*Mean*	72.0	86.4		
*SD*	27.6	19.1		
*General Health Perceptions*			150.29*	−1.09
*Mean*	56.3	75.1		
*SD*	26.8	17.3		
*Physical Component Scale*			342.95*	−1.76
*Mean*	32.7	50.0		
*SD*	16.9	10.0		
*Mental Component Scale*			35.67*	−0.48
*Mean*	45.3	50.1		
*SD*	12.6	9.9		

* Group differences at p < 0.001 according to ANOVA by group, age and gender. F-value and effectsize for the effect of group.

### Anxiety and depression

The EMWAjong group reported higher scores on the anxiety and depression scale than the reference group (p < 0.001). The differences were small to moderate with effect sizes of 0.35 and 0.54 respectively (Table 3). In addition, higher percentages (p < 0.01) of the EMWAjong group than of the reference group were *at risk* (scores ≥ 8) of disorders of anxiety (29.7 versus 17.6 percent; OR = 2.1) and depression (17.0 versus 6.0 percent; OR = 3.1) (Table 4).

**Table 3:** Anxiety and depression (HADS) of the EMWAjong group versus the HADS reference group; Mean scores, SD and effect sizes

	**EMWAjong group N = 377**	**HADS reference group N = 182**		
			**F**	**Effectsize**
*Anxiety*			12.53*	0.35
*Mean*	5.6	4.4		
*SD*	4.0	3.5		
*Depression*			18.12*	0.54
*Mean*	4.0	2.5		
*SD*	3.5	2.7		

* Group differences at p < 0.001 according to ANOVA by group and age. F-value and effectsize for the effect of group.

**Table 4:** **Proportion *at risk *(scores ≥ 8) for anxiety and depression (HADS), EMWAjong group versus the HADS reference group (Odds Ratio; OR)**

	**EMWAjong group**		**HADS reference group**		
	**%**	**N**	**%**	**N**	**OR**
*Anxiety*	29.7	112	17.6	32	2.1*
*Depression*	17.0	64	6.0	11	3.1*

* Group difference (OR) at p < 0.01 according to logistic regression analyse by group and age.

The results of the regression analyses are presented in Table 5. Respondents from the EMWAjong group who have a stable or positive course of disease reported better physical and mental HRQoL and lower levels of anxiety and depression (β = 0.46, β = 0.36, β = −.22, β = −0.22, respectively) than those with a variable or negative course of disease. In addition, those who use medical devices reported worse physical HRQoL, but better mental HRQoL and less anxiety and depression (β = −0.13, β = 0.16, β = −0.12, β = −0.22, respectively) than those without the use of medical devices. Furthermore, having a congenital disease was associated with better physical HRQoL (β = 0.13), while having a visible disease/disability was associated with worse physical HRQoL (β = −0.16).

**Table 5:** Standardized regression coefficients β for the relation of physical and mental component scale (RAND-36), anxiety and depression (HADS) with demographic and disease related variables (EMWAjong group)

	**Physical component scale**			**Mental component scale**			**Anxiety**			**Depression**		
	**B**	**SE (B)**	**β**	**B**	**SE (B)**	**β**	**B**	**SE (B)**	**β**	**B**	**SE (B)**	**β**
*Age*	−0.58	0.37	−0.07	−0.33	0.31	−0.05	0.06	0.10	0.03	−0.06	0.09	−0.04
*Female* gender 1	−3.26	1.57	−0.09*	0.57	1.29	0.02	0.06	0.42	0.01	−1.06	0.37	−0.15**
*Congenital disorder* 1	4.47	1.59	0.13**	−0.06	1.31	−0.00	−0.16	0.43	−0.02	0.09	0.37	0.01
*Perceptible disability* 1	−5.50	1.66	−0.16**	0.97	1.36	0.04	−0.48	0.49	0.06	0.25	0.39	0.04
*Stable or positive course of disease* 1	15.71	1.54	0.46**	9.20	1.26	0.36**	−1.77	0.41	−0.22**	−1.53	0.36	−0.22**
*Use of medical devices* 1	−4.48	1.64	−0.13**	3.99	1.35	0.16**	−0.91	0.44	−0.12*	−1.50	0.39	−0.22**
*F*			27,18			11,21			4,88			5,62
*R2*			0.31**			0.15**			0.07**			0.08**

^1^ coding: yes = 1, no = 0.

* p < 0.05; ** p <0.01.

## Discussion

Our hypothesis was confirmed; young adults claiming disability benefits for a childhood-onset chronic somatic condition report worse HRQoL and higher anxiety and depression scores than the reference group from the general population. Although these results may be in the expected direction and may also be in line with findings in adult populations with problems in workforce participation as a result of somatic conditions, the results are an indication of the need for support for children and adolescents who grow up with a somatic condition.

The differences in HRQoL between the EMWAjong group and the RAND-36 reference group were substantial, especially in the physical and social domains. The considerable differences in the physical domains fit the assumption that the differences in HRQoL between people with a somatic condition and healthy people are mainly based on physical limitations [23]. However, the scores on the social domain indicate that these aspects also influence the HRQoL of young adults claiming disability benefits. They may feel restricted in social situations as a result of physical or emotional consequences of their conditions. This is undesirable, especially in adolescence, because close peer relationships are an important source of support for chronically ill or disabled adolescents at a time when they have to face developmental tasks and disease-related challenges [24,25]. Research showed that the majority of the young people with a paediatric condition have peer relations and friendships that are similar to those of their peers [26]. Nevertheless, young people with visible and physically handicapping conditions may find dealing with social contexts especially difficult. Adolescents with chronic conditions may become marginalised by peers, being rejected for being different during a period in which body image and identity heavily on conformity [26,27]. The social aspects of education are a key aspect during adolescence. If the social context does not continue into a working environment due to unemployment, then young people are at risk of social isolation in later life. Therefore, it is important to encourage children and adolescents with a chronic somatic condition to make friends and to participate in social events with peers in order to build up a social life. Moreover, there is a need for preventive interventions that focus on coping skills, as they are important moderators of chronic illness effects [28,29]. In addition, guidance directed at exploring social activities which are physically feasible for the child or adolescent is recommended [26].

Even though the differences between the EMWAjong group and the general population regarding their scale scores on the Mental Health domain (one of the domains) were not significant, the EMWAjong group scored significantly worse on the summary scale scores for the overall Mental Component Scale. When we further study this aspect of the HRQoL by examining anxiety and depression, we see that the EMWAjong group scored significantly worse on anxiety as well as depression in comparison with the HADS reference group. Almost double the proportion of the EMWAjong group was at risk of an anxiety disorder, and for a depressive disorder the proportion is almost threefold. Several studies found similar results in adolescents and young adults with chronic conditions that started in childhood [30-32].

The results of the regression analyses in this study indicate that a variable or negative course of disease influences HRQoL negatively and may be a risk factor for anxiety and depression in young adult beneficiaries. This finding is in line with results of meta-analyses on anxiety and depression in children and adolescents with chronic physical illnesses [31,32]. However, due to the cross-sectional design of the study, the direction of the correlation is unknown and causality cannot be proven. The use of medical devices was found to correlate negatively with physical QoL, which we expected. However, those using medical devices reported better mental QoL as well as less anxiety and depression. The use of medical devices potentially improves patients’ psychosocial well-being regardless of their medical status. This could indicate that patients successfully adapt to their medical situation. Alternatively, the young adults benefit from the medical devices because the devices enable them to be independent, in contrast to those who do not use medical devices. Again, causality cannot be proven. Furthermore, the associations of medical devices with HRQoL, anxiety and depression were weak.

Individual differences in emotional functioning and psychological distress may be related to long-term adjustment in adulthood for young adult beneficiaries. It is still unclear which aspect – the physical or psychological part of being chronically ill or disabled – causes worse HRQoL and worse emotional well-being in young adults claiming disability benefits compared to peers from the general population. The literature on adults with chronic illness since childhood points in the same direction; a lower HRQOL and more emotional problems compared to the general population [33-36]. For this reason, and also in the light of the increasing number of young adults with a chronic disease reaching adulthood because of medical advancements [37], it is very important to pay attention to the consequences of chronic somatic conditions in an early stage. The results of this study show that paediatricians and other healthcare workers should pay attention not only to the medical but also to the emotional and psychosocial situation of patients growing up with a somatic condition. Systematic assessment of HRQoL, anxiety and depression is not yet part of standard practice, even though paediatricians and their teams know that a part of the population they treat is at risk of problems later in life. The approach in the medical context can frequently be focused on the physical consequences of the somatic condition and its treatment instead of on the patient’s emotional well-being and social life. In addition to healthcare workers and parents, it is a political and social responsibility to support children, adolescents and young adults with somatic limitations in achieving academic and vocational success. Effective support can only be addressed across systems. Cooperation between multidisciplinary rehabilitation teams and special education schools, for example, is necessary [38] in combination with the development of programmes stimulating the children and adolescents in their development. SAVTI (Successful Academic and Vocational Transition Initiative) of the Pediatric Oncology Group of Ontario (POGO) and Emma@work (job mediaton for adolescents with a somatic disease) of the Emma Children Hospital (EKZ) Academic Medical Center in the Netherlands are examples of useful tools [39].

There are a number of shortcomings of this study that need to be addressed. First, this study examined only limited number of factors influencing HRQoL, anxiety and depression and the explained variances were low. Other factors that were not examined in this study might influence psychosocial outcomes as well, for example, coping skills, personality and side effects of treatments. Also, we did control for some disease characteristics in this present study, but these characteristics merit greater attention as potentially mediating variables in predicting emotional well-being. In future research this should be addressed and more objective disease characteristics should be included. Second, our measurements and reference samples had some limitations which need to be taken into account. By choosing the RAND-36 for measuring HRQOL, differences in physical HRQoL between the EMWAjong group and the general population could be overstated because the RAND items about physical HRQoL are focused on functional limitations. Furthermore, we used two different reference samples. It should be borne in mind that the age ranges were not completely the same as the target sample and that the sample of the HADS was relatively small. Third, it is important to realise that the Wajong Act is a Dutch benefit. Most countries have no specific benefit for young disabled people [40]. Therefore, it is advisable to be cautious and conservative while interpreting results of this study and extrapolating the findings to a larger population or to other countries. Another limitation is the response rate of 20%, though this is an average response rate among young adults with a disability [41,42]. Due to the growing interest in the labour market position of young adults claiming disability benefits, they receive too many invitations to participate in all the different studies. Moreover, it is likely that respondents did not fill in the questionnaire because the invitation letter was sent by the benefits agency. Although the questionnaire was anonymous, beneficiaries might be afraid of losing their benefit. Alternatively, those with better HRQoL were less eager to participate because of reluctance to feel stigmatized. On the contrary, among those who did participate social desirability could be a threat to the validity of the results in this study. However, the reference groups used in this study consist of young adults from the general Dutch population which could also included young adults with chronic conditions. Thus, the differences in anxiety and depression are likely to be even bigger if compared with healthy peers. As a result of the need to respect the privacy of the beneficiaries, we were lacking the information regarding the non-responders to be able to pronounce upon a potential selection bias. Furthermore, the variety of chronic somatic conditions in the research population prevents the identification of high risk subpopulations within this population of young adult disability benefit recipients. It is also unknown how the group of young adults with a chronic somatic condition who apply for disability benefits compares to the group that does not apply. Therefore, the results of this study might be an underestimation or an overestimation of the problems in this group and this limits the possibility to generalize of our findings to the whole group of young adults with a chronic somatic condition. However, the problems we found in the study group are substantial and therefore socially relevant. Paying attention to this vulnerable group of young adults is of the utmost importance.

## Conclusions

The success of medical treatment in extending the lives of children with chronic conditions means that new challenges emerge. This study demonstrates worse HRQoL and increased levels of anxiety and depression experienced by young adults with disabilities or somatic illness since childhood who have to apply for disability benefits. Although some adolescents and young adults with a childhood-onset chronic somatic condition adapt well into adult life, there are many others who struggle with their overall psychosocial functioning. In medical practice, healthcare providers (including paediatric healthcare providers) should pay more attention to the HRQoL, anxiety and depression of patients growing up with a somatic condition in order to optimise their well-being and adaptation to society at the time of transition to adult life. In future research emotional functioning in young adults with a childhood-onset chronic somatic condition should be studied in more detail. Potential factors influencing HRQoL, anxiety and depression and objective disease characteristics should be taken into account in subgroup analyses in order to determine those individuals most at risk and trends within disability groups. Research is warranted to identify whether stimulating and improving job participation lead to increase of HRQoL and decrease of anxiety and depression in this group.

## Abbreviations

HRQoL: Health-related quality of life; QoL: Quality of Life; Wajong: The invalidity insurance act for young disabled persons.

## Competing interests

The authors declare that they have no competing interests.

## Authors’ contributions

EV contributed to the concept and design of the study, carried out the data acquisition, analysed and interpreted the data and drafted the manuscript. HMS contributed to the concept and design of the study, analysed and interpreted the data and drafted the manuscript. HH and MG contributed to the concept and design of the study and revised the manuscript. All authors read and approved the final manuscript.

## Authors’ information

EV is a PhD student at the Paediatric Psychosocial department of the Emma Children’s Hospital Academic Medical Center (AMC) Amsterdam. Her research examines a large cross-sectional study (EMWAjong) directed at psychosocial functioning of adolescents and young adults with disability benefits because of a chronic somatic illness or disability since childhood and at factors affecting their vocational success.

HMS is health scientist and postdoctoral researcher within the Paediatric Psychosocial department of the Emma Children’s Hospital Academic Medical Center (AMC) Amsterdam; he provides methodological support for this research.

HH is a professor in paediatrics and former Chairman of the Board of Emma Children’s Hospital Academic Medical Center (AMC) Amsterdam. He is now Chairman of the Global Health Initiative, Academic Medical Centre, University of Amsterdam.

MG is head of research of the paediatric psychology programme in the Emma Children’s Hospital Academic Medical Center (AMC) which is directed at three principal areas: studying the effects of a chronic disease or life-threatening disease on the health-related quality of life of children and young adults and family members; finding factors which predict these outcomes and development, implementation and evaluation of intervention programmes. The department has extensive research experience in coordinating randomised controlled trials of psychosocial cognitive behavioural interventions with children with chronic diseases and cancer, and developing web-based interventions for young cancer survivors and their parents.
